# Striatal ups or downs? Neural correlates of monetary reward anticipation, cue reactivity and their interaction in gambling disorder and alcohol use disorder

**DOI:** 10.1192/j.eurpsy.2023.36

**Published:** 2023-07-19

**Authors:** A. Goudriaan

**Affiliations:** Psychiatry, Amsterdam University Medical Centers, Amsterdam, Netherlands

## Abstract

**Abstract:**

Striatal dysfunction is a key characteristic of addictive disorders, but neuroimaging studies have reported conflicting findings. An integrative model of addiction points to the presence or absence of addiction-related cues as an explanation for striatal hypo- or hyperactivations, respectively, but has never been directly tested. Here, we developed a novel paradigm to investigate striatal activation during monetary reward anticipation in the presence versus absence of addiction-related pictures using functional MRI. Across two studies, we compared 24 gambling disorder (GD) patients with 22 matched healthy controls and 46 alcohol use disorder (AUD) patients with 30 matched healthy controls. A behavioral interaction was seen where gambling cues made participants respond faster for bigger, but slower for smaller rewards. During monetary reward anticipation, hypoactivation of the reward system was seen in AUD individuals compared to HCs. However, no striatal differences were seen between the participants with GD or AUD and their matched controls. In sum, these findings suggest that striatal dysfunction is a key but heterogeneous mechanism within both AUD and GD and indicate an important but complex role for addiction-related cues in explaining striatal dysfunction in addiction.

**Disclosure of Interest:**

None Declared

